# Neurobiochemical Cross-talk Between COVID-19 and Alzheimer’s Disease

**DOI:** 10.1007/s12035-020-02177-w

**Published:** 2020-10-19

**Authors:** Mohammad Azizur Rahman, Kamrul Islam, Saidur Rahman, Md Alamin

**Affiliations:** 1 Department of Biochemistry and Molecular Biology, Jahangirnagar University, Savar, Dhaka, 1342 Bangladesh; 2 Department of Chemistry, Jahangirnagar University, Savar, Dhaka, 1342 Bangladesh; 3 Global Center for Environmental Remediation (GCER), The University of Newcastle, Callaghan, NSW 2308 Australia

**Keywords:** ACE2, ApoE4, Gal-9, Inflammation, Neuroinvasive

## Abstract

COVID-19, the global threat to humanity, shares etiological cofactors with multiple diseases including Alzheimer’s disease (AD). Understanding the common links between COVID-19 and AD would harness strategizing therapeutic approaches against both. Considering the urgency of formulating COVID-19 medication, its AD association and manifestations have been reviewed here, putting emphasis on memory and learning disruption. COVID-19 and AD share common links with respect to angiotensin-converting enzyme 2 (ACE2) receptors and pro-inflammatory markers such as interleukin-1 (IL-1), IL-6, cytoskeleton-associated protein 4 (CKAP4), galectin-9 (GAL-9 or Gal-9), and APOE4 allele. Common etiological factors and common manifestations described in this review would aid in developing therapeutic strategies for both COVID-19 and AD and thus impact on eradicating the ongoing global threat. Thus, people suffering from COVID-19 or who have come round of it as well as people at risk of developing AD or already suffering from AD, would be benefitted.

## Introduction

Coronavirus disease 2019 (COVID-19) is caused by severe acute respiratory syndrome coronavirus-2 (SARS-CoV-2) that attacks predominantly the human respiratory system and has also central nervous system (CNS) targeting and neuroinvasive capabilities [1, 2]. Incubation period of SARS-CoV2 is 5 days, and the mostly noted symptoms of COVID-19 include fever, cough, and fatigue followed by or associated with headache, dyspnea, and hemoptysis [1, 2]. Acute respiratory distress syndrome, acute cardiac problems, pneumonia, and multiorgan failure had also been observed in severe cases [1, 2]. CNS manifestations in about 25% of COVID-19 patients have been reported [3]. Besides, impaired mental state, delirium, and electrolyte and metabolic derangements have been noticed in some patients [3]. Among central nervous system (CNS) comorbidities of COVID-19, Alzheimer’s disease (AD) stands first [4]. AD is a neurodegenerative disorder that affects memory and learning, behavior, and cognitive performance of the patient. The brain region (especially the hippocampus) responsible for memory and learning processes becomes affected due to deposition of amyloid beta (Aβ) or neurofibrillary tangles (NFT) in the AD patients [5]. AD symptoms appear mostly after age 60, and the patients become solely dependent on their caregivers and family members [5]. As COVID-19 management warrants isolation and quarantine, AD management does not fit with those of COVID-19 [2–4]. Thus, COVID-19 adds extra burden on AD patients, caregivers, and family members and on the national and global economy. In this regard, identification of common etiological factors would pave new vista in strategizing management and therapeutic approaches against both COVID-19 and AD. Therefore, the present review has been designed to elucidate the common links between COVID-19 and AD so that scientists, healthcare providers, policy-makers, and the general readers would be benefitted in managing the already sufferers and would also be able in safeguarding the future generation.

## SARS-CoV-2 Invasion and AD-COVID-19 Manifestations

Possible route of SARS-CoV-2 entry into the human body includes neural parenchyma, the nasal mucosa, the lamina cribrosa, retrograde axonal transport, and the olfactory bulb [6]. The neurotropism characteristic of SARS-CoV-2 aids in its invasion on the neural tissues by binding its spike protein with the angiotensin-converting enzyme 2 (ACE2) receptors present on both neurons and glial cells as well as on the capillary endothelium [6, 7]. In lungs, epithelium of the upper and lower airways harbor ACE2 mostly [6,7,8]. On the other hand, braind stem, capillary endothelium and cardiovascular function regulatory region of the CNS harbor ACE2 highly [6–8]. Compared with those of other SAR-COVs, 10–20-fold increased affinity of SARS-CoV-2 spike protein towards ACE2 has been found [8, 9]. Bypassing the ACE2 receptor, SARS-CoV-2 might utilize the olfactory bulb and avail the trans-synaptic route directly [10, 11]. Upon invasion, SARS-CoV-2 stimulates reactive astrogliosis, microglial activation, and neuroinflammatory cascade. Consequently, the blood–brain barrier (BBB) becomes compromised due to systemic inflammation followed by disrupted brain homeostasis and neuronal death [11]. Subsequent infection of the brain stem might hamper cardiovascular and respiratory regulation through chemosensory neural cells. Deranged ventilator function of the lung aggravates respiratory failure resulting in intense hypoxia [10, 11]. Combined interplay of hypoxia and neuroinflammation destroys the cortical and hippocampal structure and function, resulting in the neurological disorders. According to the direct CNS invasion proposal, SARS-CoV-2 causes inflammatory mediator release leading towards increased BBB permeability and heightened hypoxia [12]. As the CNS is devoid of the major histocompatibility antigen, it becomes solely dependent on cytotoxic T lymphocytes for removal of virus. Consequently, infectious toxic encephalopathy, acute encephalitis, and cerebrovascular attacks (CVAs) ensue [12]. Headache and seizure are symptoms of acute encephalitis; delirium and coma are symptoms of infectious toxic encephalopathy while an increased risk of CVA is a manifestation of SARS-CoV-2-provoked cytokine storm and coagulation abnormalities [12]. Neuronal expression of ACE2 escalates through nACh receptor stimulation by nicotine, and this makes the smokers much vulnerable towards neuropathological maladies [13].

### Concordant Cross-talk Between AD and COVID-19

#### Inflammo-proteomics

**Fig. 1:**
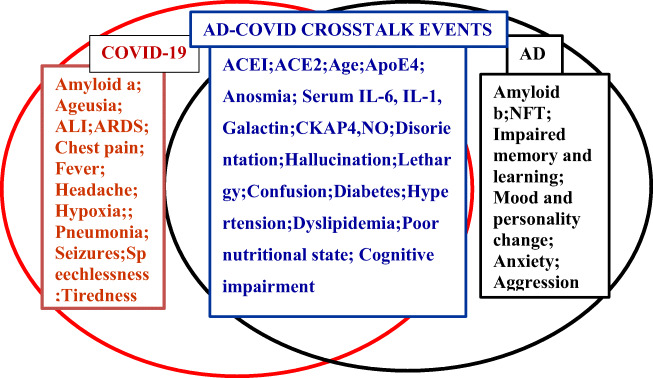
Concordant and disconcordant cross-talk between AD and COVID-19

Until recently, respiratory syndromes of SARS-COV-2 have got most attention while neurological co-manifestations have received the least though more than one-third of the patients had neurological symptoms [14]. Almost all the neurological symptoms had been manifested during the initial stage of illness [15]. Inflammatory mediators have been implicated in CNS manifestations, and immunological processes in peripheral nervous system (PNS) abnormalities, while skeletal muscle injury has been considered the direct effect of SARS-CoV-2 [10, 16, 17]. Among inflammatory markers, interleukin 6 (IL-6), interleukin 1 (IL-1), cytoskeleton-associated protein 4 (CKAP4), and galectin-9 (GAL-9 or Gal-9) had received most attention as the common links between COVID-19 and AD manifestations [18] (Fig. 1).

#### IL-6

Plasma level of inflammatory cytokines had been reported to be associated with the status of AD progression and inversely related with immune response [18]. Similarly, human cognitive performance had been inversely linked with chronic peripheral elevation of IL-6 [18]. In line with this, a significantly increased level of plasma IL-6 had been reported in 47 AD patients compared with their age-matched controls [19]. Resultant increased acute-phase proteins in the serum of AD patients are indicative of compromised immunity. Memory and learning–related behavioral tests (Morris water maze test, hole-board test, elevated plus maze test) on mice revealed that the mice deficient of IL-6 retain improved reference and spatial memory and demonstrate a better cognitive performance [20]. Though exact mechanism has not been elucidated yet, reduced IL-6 might mediate a signaling cascade involved in maintaining and restoring memory [20].

An increased serum level of IL-6 had been reported to be linked with increased COVID-19 fatality [21]. A similar trend had been linked for respiratory dysfunction [22, 23]. Plasma proteomics profiling also identified IL-6 among the most perturbed proteins in COVID-19 patients and marked as an indicator of disease severity [24]. Thus, increased serum IL-6 level is a common indicator of respiratory complications occurred in COVID-19. Also, rapid replication of SARS-CoV-2 triggers elevated production of IL-6 and heightened respiratory distress. Therefore, IL-6 stands as a common biomarker for AD and COVID-19. Antibodies capable of blocking the IL-6 receptor (tocilizumab and sarilumab) have been undergoing phase 2/3 clinical trials as the putative medications against COVID-19 [25]. As inflammatory process of AD results in neurodegeneration that could be slowed down through reduced generation of IL-6, tenidap, a non-steroidal anti-inflammatory drug, had been found promising in AD therapeutics [26]. Thus, IL-6 stands as a pleiotropic biomarker for CNS and respiratory system dysregulation among which AD and COVID-19 worth mentioning.

#### IL-1

IL-1 had been noticed to be significantly higher in the COVID-19 patients during disease onset and entire range of disease progression [27, 28]. Anakinra, a recombinant IL-1 receptor antagonist, had been found effective in improving clinical symptoms especially respiratory distress in 72% cases [27, 28]. Levels of IL-1 had also been reported to be increased in AD patients [29]. Impaired long-term potentiation and hippocampal consolidation of memory and learning processes had been associated with increased IL-1 level [30]. Injection of IL-1β in the rat brain showed increased Aβ and NFT production [31]. On the other hand, blockade of IL-1 had been found AD ameliorating [32].

#### GAL-9

Gal-9 is a β-galactoside-binding protein involved in immune reaction regulation. Its increased production had been associated with viral infection especially in the lung [33]. Thus, therapeutic strategies aimed at suppressing Gal-9 production seem pertinent in COVID-19 pandemic [34]. In the CNS, Gal-9 had been reported to be a facilitator of oligodendrocyte maturation and myelin repair mechanism [35]. Increased level of serum Gal-3 had been reported in AD patients [36]. Galectin-3 had been reported to be a promoter of Aβ oligomerization and toxicity in AD animal models [37]. Thus, galectin-3 is an inflammatory marker whose modulation seems promising in COVID-19 and AD therapeutics.

#### CKAP4

CKAP4, also known as p63, is a 63-kDa, reversibly palmitoylated and phosphorylated, type II transmembrane (TM) protein. CKAP4 regulates the quantity and survival of neuronal precursor cells (NPCs) [38]. Ablation of CKAP4 results in increased NPC death through activation of a pro-apoptotic p53-PUMA pathway as well as impaired neuronal and hippocampal memory and learning performance [38]. Though CKAP4 involvement in AD pathogenesis has not yet been reported, its role as an NPC pro-survival agent and cognitive enhancer stead this protein as a target in AD therapeutics [38]. Besides, its role in embryonic development of mammalian CNS has been regarded indispensable [38].

In lungs, CKAP4 had been implicated in maintaining lipid homeostasis through regulation of surfactant turnover [39]. In serum, lung cells, and tissues of the lung cancer patients, CKAP4 had been detected to be significantly higher than those of the healthy controls. Thus, CKAP4 stands as an early serodiagnostic marker for lung cancer and respiratory distress [40]. Plasma proteomics profiling also identified CKAP4 among the most perturbed proteins in COVID-19 patients and marked as an indicator of disease severity [24].

#### ApoE4 Allele

Apolipoprotein E is the main carrier of cholesterol in the central nervous system (CNS) and also an important constituent of very low–density lipoproteins (VLDL). Among its three alleles (ε2, ε3, and ε4), individuals carrying the ε4 allele are at a heightened risk of developing AD as the ApoE ɛ4/ɛ4 genotype increases fibrinogenesis in the brains of Alzheimer’s disease patients [41]. ApoE4 has also been reported influencing cerebral hemodynamics such as leakage of the blood–brain barrier and cerebral amyloid angiopathy [41]. Recently, APOE4 has been regarded as a marker increasing COVID-19 severity [42, 43]. Thus, AD patients carrying the APOE4 allele are at a heightened risk of developing COVID-19.

#### ACE2 Upregulation

Ten times elevated expression of ACE2 gene, SARS-CoV-2 binding protein for cell entry, had been found in the brain tissues of the AD subjects compared with those of their age-matched non-AD individuals [44]. Thus, AD patients are at a heightened risk of COVID-19 comorbidity.

#### Nitric Oxide Level

Nitric oxide (NO), an endothelium-derived relaxing factor and a neurotransmitter, plays an important role in memory and learning process and thus aids in maintaining behavioral and cognitive normalcy [45]. SARS-CoV-2, binding with the vasoconstrictor type 1 angiotensin II receptor (AT1R) through overexpressed ACE2, might lower NO production on cerebral neurons. Consequently, COVID-19 patients would become much vulnerable to behavioral and cognitive decline, the manifestations of AD [46].

#### Acetylcholine

Acetylcholine (Ach) is an excitatory neurotransmitter of the CNS and neuromuscular junction and is essential for neuronal functioning and for memory and learning abilities. According to the cholinergic hypothesis of AD, decreased availability of Ach leads towards AD consequences [47]. Produced by Ach transferase from acetyl-CoA and choline, Ach is released into the synaptic cleft and upon binding to the post-synaptic neuron, exerts signal transduction [47]. Activities of Ach are mediated through two types of receptors, namely, muscarinic and nicotinic [48]. Acetyl choline esterase (AchE) breaks down Ach and does not allow prolonged action of Ach into the post-synaptic neuron, and thus affects memory and learning abilities [47]. AD hallmarks occur due to either structural alterations in cholinergic synapses or alteration of Ach receptors or degeneration of ACh-producing neurons that ultimately lead to deteriorated cholinergic neurotransmission [47]. Therefore, treatment strategies have been developed based on this that agents having anti-AchEI activity would have ameliorating effects on AD [49]. Different AchEIs (donepezil, galantamine, rivastigmine, and tacrine) have been developed to ameliorate AD complications [49]. AchEIs have been reported to improve the cognitive and behavioral performance of the AD subjects [50].

Ach-mediated lowered production of pro-inflammatory cytokines such as tumor necrosis factor alpha (TNF-α), IL-1β, IL-6, and IL-18 and uninterrupted production of anti-inflammatory cytokine IL-10 had been reported [51]. Interestingly, AchEI galantamine had been implicated in lowering TNF-α production [52]. Therefore, inclusion of AchEIs in AD and COVID-19 therapeutics could lower the production of pro-inflammatory cytokines and aid in anti-inflammatory cytokine generation with net result: AD amelioration through Ach make-up and COVID-19 mitigation through pacification of “cytokine storm.” Another calming approach to “cytokine storm” is nicotinic receptor–mediated vagus nerve stimulation that yields cholinergic anti-inflammatory response [53, 54]. Thus, treatment strategies applying nicotinic substances and cholinergic system would shed ameliorating influence on both AD and COVID-19 [55].

#### Degenerated Cholinergic Neurons

The neurotoxic effect of Aβ oligomers deranges the cholinergic system that manifest in behavioral alteration of AD subjects [56]. Degeneration of the cholinergic neurons up to 75% in AD brains had been reported [57]. Consequent reduction in ChAT in the hippocampi and cerebral cortex of AD patients had been correlated with degenerated cholinergic nerve endings originated in the basal forebrain and septum [57]. In line with this, inverse relationship between cholinergic neurons with Aβ and NFT generation had been documented in AD animal models [58]. On the other hand, muscarinic receptor agonist or AchEI-based stimulation of the cholinergic receptor systems had been associated with shifting the amyloid precursor protein processing from amyloidogenic towards non-amyloidogenic pathway [59]. In addition to anti-inflammatory effects, stimulation of α7 nicotinic receptors had been attributed with neuroprotection against Aβ-, tau-, and NFT-induced neurotoxicity [60, 61]. Thus, treatment strategies aimed at the cholinergic system aid in amelioration of both AD and COVID-19.

#### Anosmia

Anosmia, the inability of detecting smell or taste, is a hallmark of COVID-19 [62]. Anosmia or its relevant marker hyposmia, lowered sensitivity to detect smell or taste, is also a hallmark of AD [63]. Anosmia might arise either from infection or blocked nose or due to degeneration of the nasal olfactory receptor neurons [64]. Importantly, brain injury leading to olfactory nerve or system damage may also manifest in anosmia [64]. Recently, a diminished Zn^2+^ level had been linked with COVID-19 comorbidity of anosmia [65]. SARS-CoV-2-induced local deficiency in nasal cellular zinc level might hamper the activity of Zn^2+^-dependent carbonic anhydrase, the enzyme responsible for olfaction. Immunologically, depleted Zn^2+^ level might shift the Th1/Th2 balance to Th2 predominance resulting in increased IL-6 generation of COVID-19 subjects [65]. In this connection, decreased blood Zn^2+^ level had been associated with AD [66]. Cognitive impairment associated with olfactory dysfunction had become a common marker of AD and COVID-19.

### Discordant Cross-talk Between AD and COVID-19

Besides the abovementioned similarity-based cross-talks, there exists some disparity-oriented discourse between AD and COVID-19 [67] (Fig. 1). For example, headache, cough, and seizures are common features of COVID-19 but not of AD [67] (Fig. 1). Some other contrasting features are as follows:

#### Age

Older people are at a higher risk of falling victim of both AD and COVID-19 [68]. But, for patients aged over 80 years, further aging is not a risk factor for COVID-19, rather for dementia and AD [68]. Though the exact mechanism is not clear yet, reduced susceptibility of secondary lung inflammation might be the cause [68]. On the other hand, AD susceptibility usually begins on or after 60 years of age and as aging advances, so soars the AD pathogenesis [69]. Heightened production of reactive oxygen species (ROS), exacerbated amyloid beta production, aggregation and neurodegeneration, perturbed proteostasis, cardiovascular diseases (CVD), diabetes, hypertension, and lifestyle modification had been implicated in AD pathogenesis of the aged persons [69].

#### Sex

Compared with females, males had been found much vulnerable to COVID-19 fatality [70]. Increased ACE2 level, effect of testosterone on ACE2, imbalance among ACE2 products (Ang 1–7, Ang 1–9), and dire onslaught of cytokine storm are among the possible factors affecting men much than those of the women [70]. Thus, manipulation of ACE2 expression through sex hormone modulators seems pertinent in treating COVID-19. On the other hand, estrogen and testosterone levels had been found neuroprotective and amyloid beta–clearing agents [71]. In female AD patients older than 80 years, brain levels of androgen and estrogen had been found lower than their age-matched non-AD counterparts [72]. In case of normal and AD male subjects, the downtrend level of androgen and testosterone had been observed as aging progresses over 70 years [72, 73]. Thus, disparity in sex hormone levels contributes to the biased prospect of AD or COVID-19 in men and women. Keeping pace with this fact, treatment strategies might be formulated to restore the sex hormone levels in respective patients.

#### Different Treatment Strategies

AD and COVID-19 differ in their etiology. AD is caused by deposition of abnormally higher levels of Aβ or NFT. Thus, treatment strategies against AD focus mainly withstanding Aβ production or accelerating its clearance [74]. On the other hand, COVID-19 is caused by SARS-CoV-2 entry into host cell and subsequent inflammatory, respiratory, cardiovascular, CNS, and psychological complications. Thus, COVID-19 treatment strategies tend to impede viral entry, viral replication, and subsequent symptom amelioration. In this regard, SARS-CoV-2-directed drugs (remdesivir, lopinavir), host-targeting agents such as ACE/ACE2 receptor inhibitors, angiotensin receptor blockers (ARB), and immunomodulators such as inhibitors to IL-6 and IL-1, and convalescent plasma therapy had been in practice worldwide [75].

## Conclusion

In addition to the persisting COVID-19 complications, its long-term consequences have been shaking the healthcare professionals globally. Alzheimer’s disease stands among the top-notch out-turn of COVID-19. Etiological cofactors and physiological co-manifestations described in this review would succor in strategizing therapeutic approaches against both COVID-19 and AD. We must admit that we have depended only upon the data available at hand, and we must look towards future directions from the scientific community to hold back the global crises like COVID-19 and AD.

## Data Availability

Not applicable.
